# Management of a case of high-risk gastrointestinal stromal tumor in rectum by transanal minimal invasive surgery

**DOI:** 10.1186/s12957-018-1463-x

**Published:** 2018-08-11

**Authors:** Pramod Nepal, Shinichiro Mori, Yoshiaki Kita, Kan Tanabe, Kenji Baba, Yasuto Uchikado, Hiroshi Kurahara, Takaaki Arigami, Masahiko Sakoda, Kosei Maemura, Shoji Natsugoe

**Affiliations:** Department of Digestive Surgery, Breast and Thyroid Surgery, Graduate School of Medicine, Kagoshima University, Sakuragaoka 8-35-1, Kagoshima, 890-8520 Japan

**Keywords:** Rectal GIST, TAMIS, Imatinib mesylate

## Abstract

**Background:**

Rectal gastrointestinal stromal tumor (GIST) is a very rare tumor of gastrointestinal tract. Surgical management of rectal GIST requires special attention for preserving of anal and urinary functions. Transanal minimal invasive surgery (TAMIS) is a well-developed minimally invasive technique for local excision of benign and early malignant rectal tumors; however, the application of TAMIS for rectal GIST is rarely and inadequately reported. We report the novel application of TAMIS for rectal GIST with considerations for anal and urinary functions.

**Case presentation:**

A 67 years old female, who presented with history of per rectal bleeding, was diagnosed with submucosal GIST of 4.5 cm in diameter at right posterior wall of 7 cm from anal verge. Histology of biopsy showed abundant spindle-shaped cells arranged in bundles that were positive for CD34 and negative for C-Kit, desmin, smooth muscle actin (SMA), and S-100. The tumor was excised by TAMIS successfully. Final histopathology showed pT2 tumor with C-Kit positive and mitosis count 10 per 50 HPF. Postoperative period was uneventful, and she was discharged on adjuvant imatinib mesylate for 3 years.

**Conclusion:**

TAMIS can be used safely in the management of rectal GIST after appropriate evaluation of tumor size, extent, location, and experience of operating surgeon.

## Background

Gastrointestinal stromal tumor (GIST) is a rare tumor of the gastrointestinal (GI) tract that constitutes less than 1% of all GI tumors. Nevertheless, they are the commonest of all the mesenchymal tumors of the GI tract [1]. The usual sites of occurrence are the stomach (60–70%), intestines (20–30%), colon and rectum (5%), and esophagus (< 5%) [2]. GISTs in the rectum demonstrate male predominance and rarely occur in individuals younger than 40 years [3].

Various surgical techniques have been described for the treatment of rectal GIST, including traditional transanal resection, trans-sacral approach, transanal endoscopic microsurgery (TEM), transanal minimal invasive surgery (TAMIS), and laparoscopic surgery [4–11]. Although TAMIS has been undergoing a surge in popularity among surgeons, its application for management of rectal GISTs is rarely reported, and only few cases of rectal GISTs are included in large series of TAMIS [5, 6]. Here, we discuss a high-risk case of rectal GIST that was managed by TAMIS with due consideration for preserving anal and urinary functions and by postoperative adjuvant therapy with imatinib mesylate (IM).

## Case presentation

**Fig. 1:**
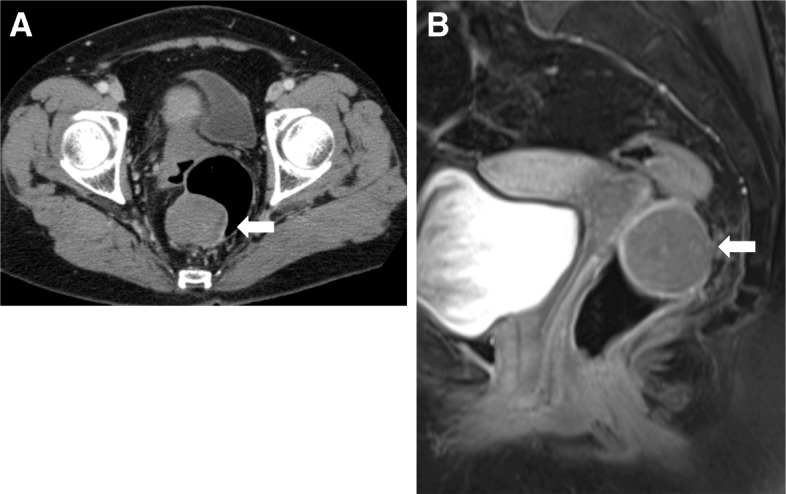
CT and MRI examinations. **a** CT scan of pelvis showing tumor 4.5 cm in diameter (white arrowhead). **b** MRI showing tumor at middle rectum (white arrowhead)

**Fig. 2:**
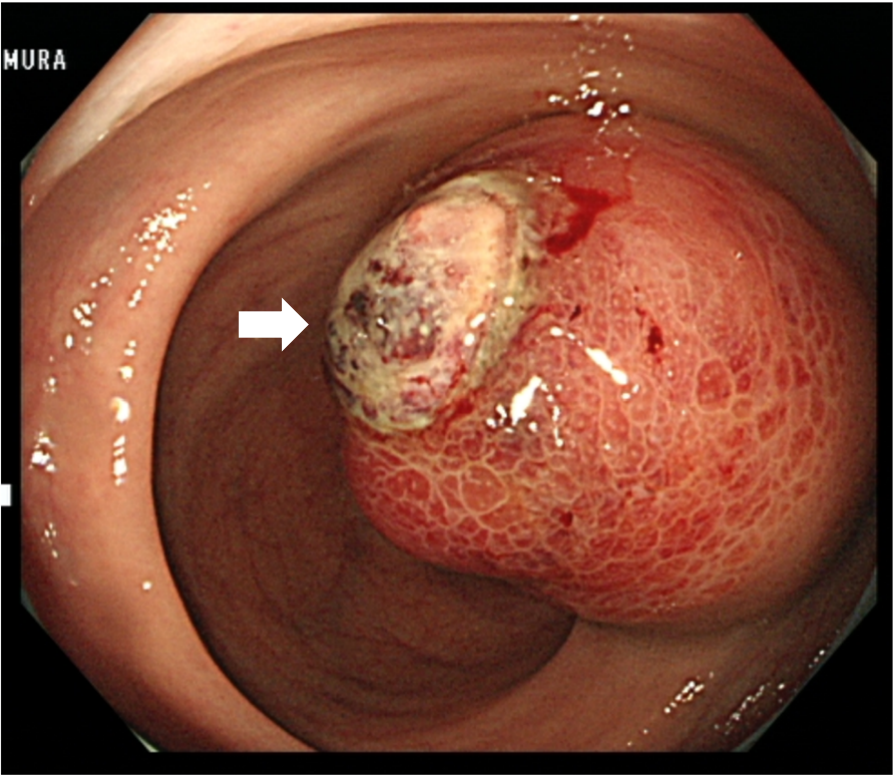
Colonoscopic image showing tumor with ulcer in right lateral wall of middle rectum (arrowhead)

**Fig. 3:**
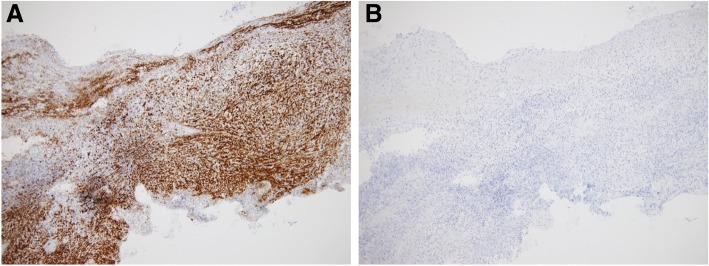
Histopathology of biopsy. **a** The biopsy was positive for CD34. **b** The biopsy was negative for C-kit

A 67-year-old female patient presented with a complaint of per rectal bleeding. Computed tomography (CT) and magnetic resonance imaging (MRI) showed a tumor 4.5 cm in diameter in right posterior wall of the middle rectum with no adjacent infiltration or lymph node metastasis (Fig. 1a, b). Colonoscopy revealed a submucosal mass in the right posterior wall of the middle rectum 7 cm from anal verge (Fig. 2). Histologically, a biopsy showed spindle-shaped cells arranged in bundles, positive for CD34 and negative for C-Kit, Desmin, smooth muscle actin, and S-100 (Fig. 3). These findings suggested a rectal GIST, and TAMIS was scheduled.

**Fig. 4:**
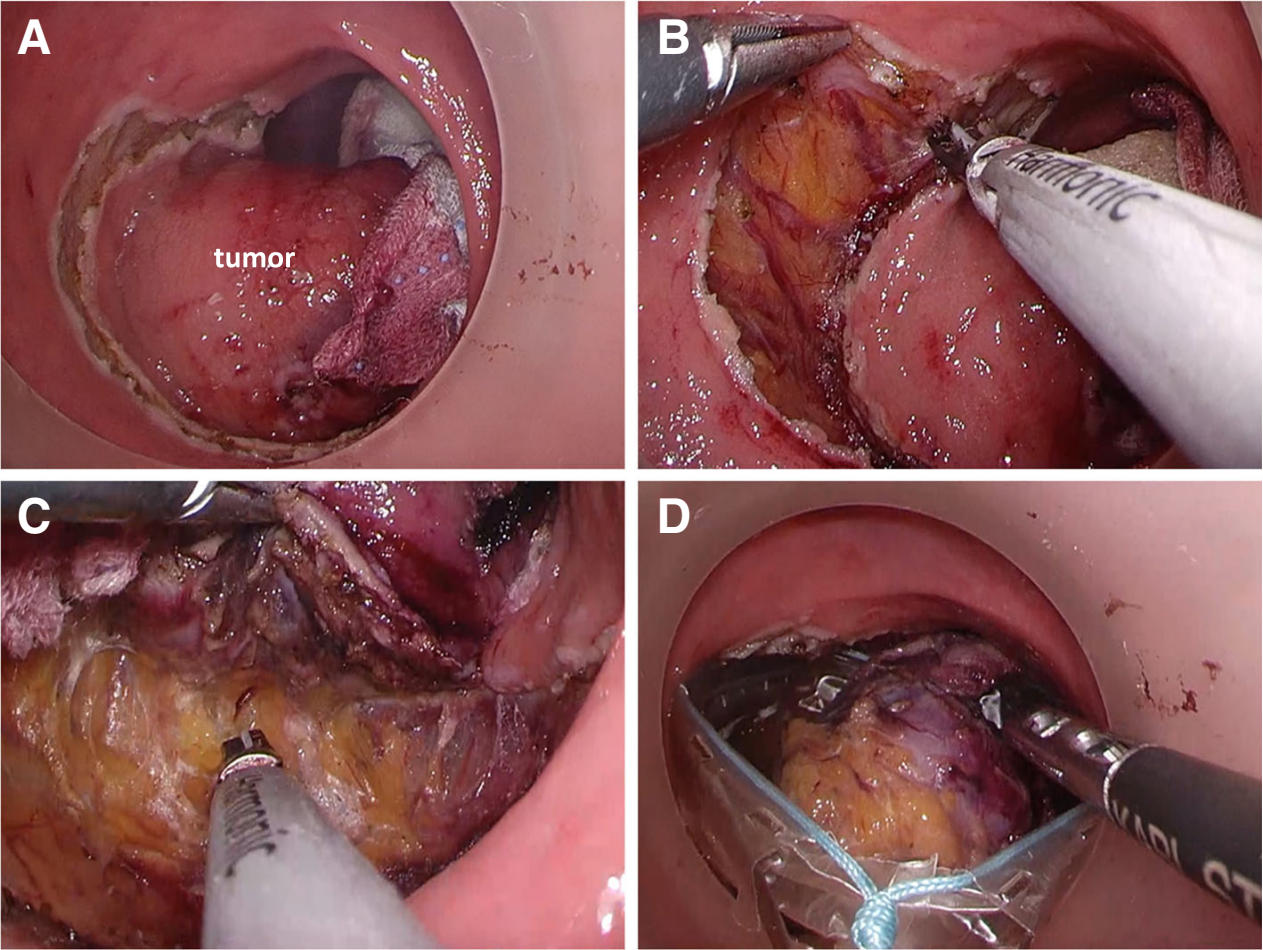
Surgical procedure. **a** Mucosal dissection performed along the tattoo circumferentially 1 cm from the tumor margin. **b** Full-thickness dissection of tumor performed circumferentially. **c** Dissection performed between rectum and mesorectum on the posterior wall. **d** Extraction of the tumor using the Endo Catch specimen pouch

**Fig. 5:**
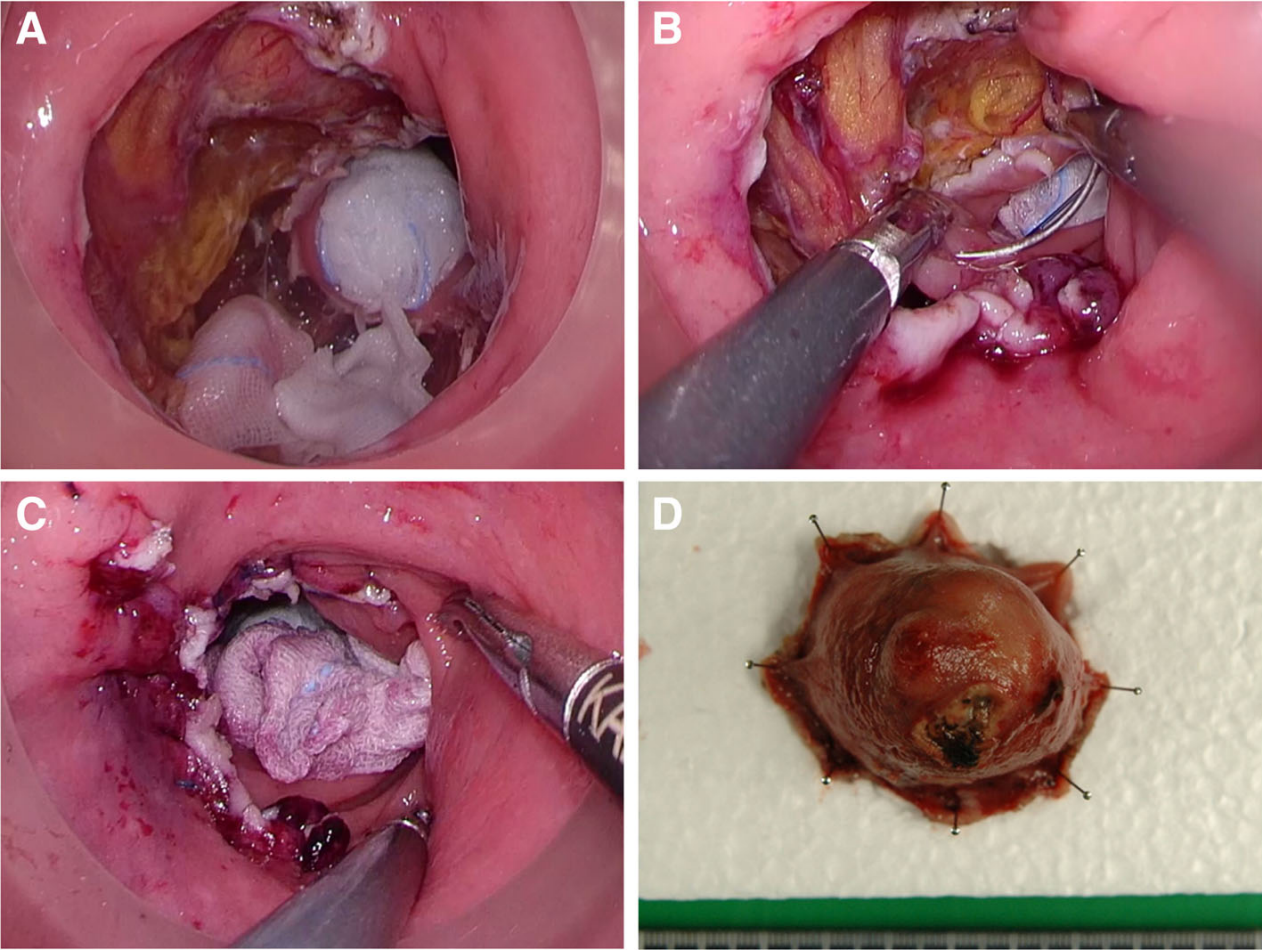
Defect closure and retrieved specimen. **a** Intraluminal lavage with saline and securing hemostasis. **b** Closing the defect with 3-0 V-Loc under 8 mmHg pressure under the AirSeal system. **c** Final view of surgical site after repair of rectal defect (LDQ). **d** The specimen measuring 4.5 cm × 4.5 cm × 3.5 cm

The patient was kept in the modified lithotomy position, and the anus dilated with a self-retaining anal retractor (Lone Star Retractor; Cooper Surgical, Trumbull, CT, USA). A transanal access device (GelPOINT path; Applied Medical, Rancho Santa Margarita, CA, USA) was introduced. Wet gauze was inserted above the lesion, and pneumorectum was maintained at 15 mmHg with carbon dioxide by an AirSeal platform (AirSeal system; CONMED, Utica, NY, USA). Conventional laparoscopic instruments were used. The tumor was located at the right posterior wall in the middle rectum; the incision site 1 cm away from the tumor margin was tattooed circumferentially. Mucosal dissection was performed along the tattoo (Fig. 4a), and subsequent full-thickness excision was carried out (Fig. 4b, c). The tumor was peeled off and extracted using an Endo Catch specimen pouch (Medtronic, Minneapolis, MN, USA) to avoid dissemination (Fig. 4d). Intraluminal lavage with saline was performed, and hemostasis was secured (Fig. 5a). The defect was closed with 3-0 V-Loc (Medtronic) under 8 mmHg pressure by the AirSeal system (Fig. 5b, c). The specimen measuring 4.5 cm × 4.5 cm × 3.5 cm (Fig. 5d) was sent for histopathology, which confirmed a pT2 rectal GIST positive for KIT (CD117) and CD34. The resection margin was negative, and the mitosis count was 10 per 50 high-power fields. The postoperative period was uneventful with normal anal and urinary functions. The patient was discharged on IM (Gleevec), 400 mg once daily for 3 years under regular follow-up.

## Discussion

Surgery with complete resection is the only curative option for rectal GISTs [1]. It is very important to consider the balance of radical resection with the preservation of the anal and urinary functions in the treatment of middle to lower rectal GISTs. Various surgical techniques have been described for rectal GISTs, including conventional transanal resection, trans-sacral approach, transanal endoscopic microsurgery (TEM), transanal minimal invasive surgery (TAMIS), and laparoscopic surgery [4–11]. Clinicians need to adopt these approaches according to appropriate evaluation of tumor size, extent, and location, as well as the operating surgeon’s experience of the techniques.

TEM, which had better access to proximal rectum and good surgical field of vision, provides superior quality of resection, decreased local recurrence compared to conventional transanal excision for selected patients with rectal lesions [12, 13]. Compared with the abdominoperineal resection (APR) and some other function-preserving procedures, TEM also is much more minimally invasive with less morbidity and better life quality for selective patients [12, 13]. However, TEM has been slow to become universally adopted by colorectal surgeons, in part because of a steep learning curve, but also because of the significant cost of the highly specialized instrumentation [14–16]. TAMIS was first reported by Atallah as a hybrid between TEM and single-port laparoscopy in 2010, who concluded that TAMIS is a feasible alternative to TEM, providing its benefits at a fraction of the cost [16]. We adopted TAMIS using GelPOINT path as a surgical management of our case. The technique yielded good visualization of operation field and allowed precisely full thickness excision of the tumor with preserving mesorectum, resulting in preserving urinary function. However, when patients have a tumor larger than 5 cm in diameter or very close to the anal verge, the adoption of laparoscopic APR or other function-preserving procedures is unavoidable. Table 1 shows the summary of surgical options for resection of rectal GIST. In our case, TAMIS was able to be applied because the tumor was 4.5 cm in diameter with no metastasis and was located in the mid-rectum. Postoperatively, the patient had satisfactory anal and urinary function, with no recurrence or metastasis at the 12-month follow-up.

**Table 1:** Summary of surgical procedures for the resection of rectal GIST

S.N	Procedure	Benefits	Demerits	Cost	Morbidity rate.
1.	Local trans-anal resection [6, 12]	• Used usually for lower rectal lesions • Easy and minimally invasive	Local recurrences is high due to poor quality of excision and fragmentation of tumor	Cheaper	Up to 22%
2.	Trans-sacral resection [11, 20]	Beneficial for GISTS that are large and grow away from rectal lumen	• More invasive than TAMIS • Increased risk of poor perineal wound healing and fecal fistula	Cheaper	Up to 21%
3.	TEM [12, 16]	Superior quality of resection, decreased local recurrence, and improved survival	• Anorectal dysfunction may occur due to rigid anoscope • Steep learning curve and need of highly qualified surgeon	Expensive than TAMIS	Up to 29%
4.	TAMIS [6, 16]	• Superior operative results • Convenient access device and less effects on anorectal functions	Difficult to access upper rectum and not suitable for large tumors	Reasonable cost	Up to 7.4%

With surgery alone, the 15 years recurrence-free survival (RFS) and overall survival (OS) time was found to be 59.9% and 12.4 years respectively [17]. Use of IM as an adjuvant therapy can increase resectability or decrease surgical morbidity in unresectable or locally advanced cases and can improve recurrence-free survival [4]. The tumor size, mitosis count, non-gastric location, male sex, and rupture of pseudocapsule are the independent adverse prognostic factors for GIST [17]. In our case, the tumor diameter was 4.3 cm and initial biopsy was negative for C-Kit, so we proceeded with curative surgery rather than neoadjuvant IM. Neoadjuvant IM is recommended if R0 resection is not possible, surgery can be achieved by less mutilating surgery/functional preserving surgery, or can be made safer [18]. In our case, the mitosis count was 10 per 50 HPF, so it was considered high-risk malignant GIST [19] and patient was discharged on adjuvant IM for 3 years.

This case provided a new strategy consisting of TAMIS using GelPOINT path with conventional laparoscopic instruments for patients with small size of tumor less than 5 cm in diameter. However, prospective studies are needed in the future to investigate safety and effects of this new strategy.

## Conclusion

Rectal GIST is one of the most important differential diagnoses of rectal tumor that requires special consideration with regard to preservation of anal and urinary functions when the tumor is small. In our case, TAMIS using GelPOINT path contributed to curative resection of the tumor and satisfactory functions. The appropriate surgical technique should be selected depending upon location, size, and resectability of tumor, and the surgical expertise of the attending physician.

## Abbreviations

APR: Abdominoperineal resection
CT: Computed tomography
GI: Gastrointestinal
GIST: Gastrointestinal stromal tumor
IM: Imatinib mesylate
MRI: Magnetic resonance imaging
RFS: Recurrence-free survival
TAMIS: Transanal minimal invasive surgery
TEM: Transanal endoscopic microsurgery
